# A Comparison of the Antitumor Efficacy of Novel Multi-Specific Tribodies with Combinations of Approved Immunomodulatory Antibodies

**DOI:** 10.3390/cancers15225345

**Published:** 2023-11-09

**Authors:** Lorenzo Manna, Rosa Rapuano Lembo, Asami Yoshioka, Koji Nakamura, Margherita Passariello, Claudia De Lorenzo

**Affiliations:** 1 Department of Molecular Medicine and Medical Biotechnology, University of Naples “Federico II”, Via Pansini 5, 80131 Naples, Italy; lorenzo.manna@unina.it; 2 Ceinge—Biotecnologie Avanzate s.c.a.r.l., Via Gaetano Salvatore 486, 80145 Naples, Italy; rosa.rapuano@unimi.it; 3 European School of Molecular Medicine, University of Milan, 20122 Milan, Italy; 4 Chiome Bioscience Inc., 3-12-1 Honmachi Shibuya-Ku, Tokyo 151-0071, Japan; asyoshioka@chiome.co.jp (A.Y.); knakamura@chiome.co.jp (K.N.)

**Keywords:** immunotherapy, cancer, immunomodulatory mAbs, bispecific antibodies, PD-L1, LAG-3, combinatorial treatments

## Abstract

**Simple Summary:**

One of the main challenges faced by cancer immunotherapy is the emergence of resistance against monotherapies, mediated by several immune escape mechanisms, which are able to impair therapy outcomes. Here, we investigated the biological properties of two novel bispecific constructs, known as tribodies, previously generated in our laboratory. These two bispecific molecules, named TR0304 and TR0506, are able to bind simultaneously to two immune checkpoints, i.e., LAG-3 and PD-L1 or LAG-3 and PD-1, respectively. After testing their effects on lymphocytes and on co-cultures of immune and tumor cells by comparing them to the monoclonal antibodies currently approved for clinical use, we found that these new constructs were able to activate the hPBMCs more efficiently than clinically validated mAbs, leading to stronger tumor cell lysis and, thus, showing their therapeutic potential for monotherapy-resistant tumors.

**Abstract:**

Many advances in antitumor therapies have been achieved with antagonistic antibodies targeting the programmed cell death protein 1 (PD-1) or its ligand (PD-L1); however, many cancer patients still develop resistance to anti–PD-1/PD-L1 treatments often associated with the upregulation of other immune checkpoints such as Lymphocyte Activation Gene-3 (LAG-3). In order to verify whether it is possible to overcome these limits, we analyzed and compared the effects of combinations of the clinically validated anti-LAG-3 mAb (Relatlimab) with anti-PD-1 (Pembrolizumab) or anti-PD-L1 (Atezolizumab) monoclonal antibodies (mAbs) with those of novel bispecific tribodies (TRs), called TR0304 and TR0506, previously generated in our lab by combining the binding moieties of novel human antibodies targeting the same ICs of the mentioned mAbs. In particular, TR0304, made up of a Fab derived from an anti-PD-L1 mAb and two single-chain variable fragments (scFvs) derived from an anti-LAG-3 mAb, was tested in comparison with Relatlimab plus Atezolizumab, and TR0506, made up of an antigen-binding fragment (Fab) derived from the same anti-LAG-3 mAb and two scFvs derived from an anti-PD-1 mAb, was tested in comparison with Relatlimab and Pembrolizumab. We found that the two novel TRs showed similar binding affinity to the targets with respect to validated mAbs, even though they recognized distinct or only partially overlapping epitopes. When tested for their functional properties, they showed an increased ability to induce lymphocyte activation and stronger in vitro cytotoxicity against tumor cells compared to combinatorial treatments of clinically validated mAbs. Considering that tribodies also have other advantages with respect to combinatorial treatments, such as reduced production costs and lower dose requirements, we think that these novel immunomodulatory TRs could be used for therapeutic applications, particularly in monotherapy-resistant cancer patients.

## 1. Introduction

Immunotherapy is an emerging approach in cancer treatment, and it is rapidly becoming one of the most prominent research areas in the oncology field. This growing trend can be easily explained not only by the proven efficacy of this type of treatment against different tumors but also as a result of the notably lower side effects observed in comparison with the conventional approaches in cancer therapy, namely, radiotherapy and chemotherapy [1,2,3]. Cancer cells exploit a plethora of immune escape mechanisms in order to elude the immune system response [4,5]; thus, one of the goals of immunotherapy is to induce a stronger response in immune cells against malignant ones. One of the most effective strategies to reach this goal is the blockade of immune checkpoints (ICs).

Immune checkpoints are molecules able to transduce negative regulatory signals affecting immune system cell activity, thereby reducing their ability to identify and kill cancer cells. The pathways regulated by these molecules are physiologically involved in self-tolerance mechanisms, which are essential to avoid the excessive activation of immune cells, which could lead to tissue damage. Malignant cells can use these molecules to their advantage in order to dampen the immune response, leading to their uncontrolled proliferation [6,7]. Several molecules have been identified and classified as immune checkpoints over the years; among these, Programmed cell Death protein 1 (PD-1) receptor and its ligand Programmed Death-Ligand 1 (PD-L1), together with Lymphocyte Activation Gene 3 (LAG-3), have been extensively studied and have proven to be effective immunotherapy targets. The pathway regulated by PD-1 inhibits several transcription factors, such as the nuclear factor-κB (NF-κB) and the nuclear factor of activated T cells (NFAT); this pathway is triggered when PD-1 is engaged by PD-L1, which is usually overexpressed in tumor cells [6,8,9]. Similarly, LAG-3 inhibition showed an improved activation of both CD8+ and CD4+ T cells, while also affecting Treg cells’ activity [6,10,11,12].

The most effective way to block the activity of these immune checkpoints, thereby leading to enhanced immune system-neutralizing activity, is through antagonistic monoclonal antibodies (mAbs). During the last decade, a great number of monoclonal antibodies have been generated and tested in vitro and in vivo for their ability to counteract the immune escape of tumor cells, and some of them have been approved by the FDA for clinical use in different types of tumors, for instance, Atezolizumab (an anti-PD-L1 mAb) for the treatment of non-small-cell lung cancer (NSCLC), Pembrolizumab (an anti-PD-1 mAb) for triple-negative breast cancer (TNBC), and, more recently, Relatlimab (an anti-LAG-3 mAb) for metastatic or unresectable melanoma [13,14,15].

In the past few years, we have generated a wide range of human monoclonal antibodies, using an innovative phage display strategy on activated lymphocytes, followed by screening on purified target proteins and next-generation sequencing (NGS). This screening process was performed for several immune checkpoints, and three monoclonal antibodies in particular, named PD-1_1, PD-L1_1, and LAG-3_1, were isolated and extensively characterized, recognizing PD-1, PD-L1, and LAG-3, respectively [16]. These antibodies showed different intriguing properties: they were able to bind to their targets with comparable or even higher affinity than the clinically validated mAbs, and they were found to be capable of more efficiently activating human peripheral blood mononuclear cells (hPBMCs), resulting in increased cytokine secretion. Indeed, when the antibodies were used in co-cultures of hPBMCs with tumor cells, they showed more potent tumor cell lysis when compared to the mAbs approved for clinical use, and these effects were enhanced when the antibodies were used in combinatorial treatments [16,17,18,19].

On the basis of these promising results, we decided to generate new constructs, called tribodies (TRs), in an attempt to combine the biological properties of two different antibodies into one single molecule. These innovative constructs are made up of a Fab fragment, recognizing one immune checkpoint, and two single-chain variable fragments (scFvs), recognizing a second different immune checkpoint. In particular, two of them, called TR0304 and TR0506, are made up of a Fab fragment specific for either PD-L1 or LAG-3, and two scFvs are derived from either LAG-3_1 or PD-1_1 mAbs, respectively. These new tribodies retained the binding ability of the parental mAbs; they were also capable of more efficiently activating hPBMCs and, subsequently, inducing stronger tumor cell lysis in co-culture assays than the parental mAbs, even when the latter were used in combination [20].

Considering the frequent emergence of resistance to monotherapy in the tumor microenvironment due to the compensatory expression of other ICs [21,22], a construct combining two different binding moieties against two distinct ICs could be an attractive strategy to overcome tumor resistance to monotherapies. Here, we further characterized the biological properties of these newly generated tribody constructs by performing a comparative analysis with the clinically validated mAbs, used alone or in combination, in order to evaluate whether the tribodies could potentially provide an efficient alternative in immunotherapy-based treatments.

## 2. Materials and Methods

### 2.1. Antibodies and Human Recombinant Proteins

The following commercial and clinically validated antibodies were used: anti-PD-L1 Atezolizumab mAb (InvivoGen, San Diego, CA, USA); anti PD-1 Pembrolizumab (Merck Sharp & Dohme B.V., Haarlem, The Netherlands); anti-LAG-3 Relatlimab (Bristol-Myers Squibb, Princeton, NJ, USA); commercial human anti-PD-L1 (G&P Biosciences, Santa Clara, CA, USA); anti-human IgG (Fab’)2 goat HRP-conjugated monoclonal antibody (Abcam, Biomedical Campus, Cambridge, UK); and HRP-conjugated 6*His, His-Tag Monoclonal antibody (Proteintech, Rosemont, IL, USA).

The following recombinant proteins were used: Recombinant Human LAG-3 Fc Chimeric Protein, Recombinant Human PD-L1/B7-H1 Fc Chimeric Protein, and Recombinant Human PD-1 Fc Chimeric Protein (all from R&D Systems, Minneapolis, MN, USA)

### 2.2. Tumor Cell Cultures

The human triple-negative breast cancer (TNBC) MDA-MB-231 and BT-549 cell lines and non-small-cell lung cancer (NSCLC) A-549 cell line were obtained from the American Type Culture Collection (ATCC, Rockville, MD, USA). MDA-MB-231 cells were cultured in Dulbecco’s Modified Eagle’s Medium (DMEM, Gibco, Life Technologies, Paisley, UK). BT-549 cells were cultured in Roswell Park Memorial Institute 1640 Medium (RPMI 1640, Gibco, Life Technologies, Paisley, UK). A-549 cells were cultured in Kaign’s Modification of Ham’s F-12 Medium (F-12 K Gibco, Life Technologies, Paisley, UK). The media were supplemented with 10% (vol/vol) heat-inactivated fetal bovine serum (FBS Euroclone S.p.A., Pero, Milano, Italy), 50 U/mL penicillin, 50 μg/mL streptomycin, and 2 mM L-glutamine (all from Gibco Life Technologies, Paisley, UK) and cultured in a humidified atmosphere of 95% air and 5% CO_2_ at 37 °C.

### 2.3. The Isolation of Human Peripheral Blood Mononuclear Cells

Human peripheral blood mononuclear cells (PBMCs) were isolated from the blood of healthy donors using a Greiner Leucosep^®^ tube (Sigma-Aldrich, St. Louis, MO, USA) following the manufacturer’s instructions, as previously reported [16,19,23,24,25], and frozen in 90% FBS plus 10% dimethyl sulfoxide (DMSO) solution until use. Cryopreserved cell vials were gently thawed using RPMI 1640 medium supplemented with 1% L-glutamine, 1% CTLWash™ (Immunospot by Cellular Technology Limited, Shaker Heights, Cleveland, OH, USA) and 100 U/mL Benzonase (Merck Millipore, Darmdstadt, Germany) and washed via centrifugation. The collected lymphocytes were incubated overnight at 37 °C in R10 medium (RPMI 1640 supplemented with 10% inactivated FBS, 1% L-glutamine, 50 U/mL penicillin, 50 μg/mL streptomycin, and 1% HEPES (Gibco, Life Technologies, Paisley, UK) before their use for functional assays.

### 2.4. The Production and Purification of the Tribodies

**Figure 1:**
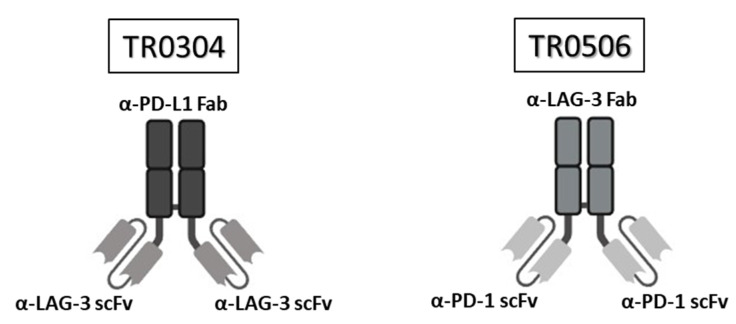
Structure of the tribodies TR0304 and 0506. Schematic representation of the bispecific tribodies TR0304 and TR0506 targeting LAG-3 and PD-L1 or PD-1, respectively. Each TR was obtained by genetically fusing the indicated Fab specific for one IC with two identical scFvs specific for the second IC.

The tribodies TR0304 and TR0506, reported in Figure 1, were produced as previously described [20] in collaboration with the “Chiome Inc” company (Tokyo, Japan) by using the Trisoma platform technology to express and purify the constructs. The sequences encoding the anti-PD-L1 or anti-LAG-3 fab and the anti-PD-1 or anti-LAG-3 scFvs inserted in the novel generated tribodies were previously reported (PCT/EP2019/057239) [16]. The tribodies were obtained by co-transfecting Expi293 cells with expression vectors encoding the heavy and light chain of each construct and purified from the medium via affinity chromatography by using His-tag purification resin, as described in [20]. Endotoxin analysis was performed using a LAL-based method. The tribodies were filtered, aliquoted, and tested for their stability before and after storage at −70 °C via SDS-PAGE and size-exclusion chromatography (SEC) analysis.

### 2.5. Enzyme-Linked Immunosorbent Assays (ELISAs)

To test the binding of Atezolizumab, Pembrolizumab, Relatlimab, or the tribodies (TR0304 and TR0506) to their target expressed on hPBMCs, cell ELISAs were performed as previously described [25]. The cells, previously activated with anti-CD-3/CD-28 beads (25 µL/1 × 10^6^ cells, following the manufacturer’s recommendations (Gibco, Life Technologies, Paisley, UK)), were plated in triplicates into a Nunc round-bottom 96-well plate in suspension at a density of 2 × 10^5^ cells/well and incubated with a blocking solution (PBS/BSA 6%) for 20 min at RT. After blocking, lymphocytes were recovered via centrifugation at 1200 rpm for 10 min at room temperature (RT). Then, the cells were incubated in the absence or presence of Atezolizumab, Pembrolizumab, Relatlimab, or the tribodies at increasing concentrations (ranging from 0.01 nM to 100 nM) in PBS/BSA 3% buffer solution for 2 h at RT with gentle agitation. The incubation with the primary antibodies was followed by three washes with PBS 1× and, then, incubation with an appropriate HRP-conjugated secondary antibody for 1 h at RT. Later, the plate was washed and 3,3’,5,5’-Tetramethylbenzidine (TMB) (Sigma-Aldrich, St. Louise, MO, USA) reagent was added for 10 min before quenching with an equal volume of 1 N HCl. The absorbance at 450 nm was measured with an Envision plate reader (Perkin Elmer, 2102, San Diego, CA, USA).

### 2.6. Biolayer Interferometry (BLI) Analyses

The BLI analyses were performed by using the Octet^®^ R4 Protein Analysis System (Sartorius, Fremont, CA, USA). Biosensors carrying the protein A (Octet^®^ ProA Biosensors, Sartorius, Fremont, CA, USA) were used to perform the assays. Prior to the BLI run, the ProA biosensors’ tips were hydrated for 15 min in 200 µL of Kinetic Buffer (KB) 10× (0.1% BSA, 0.02% Tween, in PBS 1×). Then, the program steps were set up using the BLI software (Octet^®^ BLI Discovery Software 13.0). The biosensors were loaded with recombinant human LAG-3 Fc, PD-L1 Fc, or PD-1 Fc (all from R&D Systems, Minneapolis, MN, USA) used at a concentration of 2 µg/mL, for a time interval of up to 500 s. After washing, the association step was carried out by incubating the biosensors for 600 s in a solution containing the analytes (TR0304, TR0506, Atezolizumab, Pembrolizumab, or Relatlimab) diluted at increasing concentrations (1, 10, and 100 nM). The dissociation step was performed in KB buffer 10 for 200 s. For epitope binning analyses, after washing, each ligand was saturated with Atezolizumab, Pembrolizumab, or Relatlimab at a concentration of 200 nM for 600 s; then, the TR0304 or TR0506 tribodies were added at increasing concentrations (50 nM, 100 nM, and 200 nM) for 600 s. Finally, the biosensors were regenerated according to the manufacturer’s recommendations. The data were acquired and processed into the Octet^®^ Analysis Studio Software 13.0.

### 2.7. Cytokine Secretion Assays

The supernatants of the treated hPBMC cultures or co-cultures of tumor cells with hPBMCs were analyzed using ELISAs to evaluate the secretion of interleukin-2 and IFN-γ. Briefly, after the treatments in the absence or presence of immunomodulatory mAbs, their combination, or the tribodies at the indicated concentrations, the supernatants were centrifuged and treated for the quantification of human IL-2 and IFN-γ (DuoSet ELISA, R&D Systems, Minneapolis, MN, USA), according to the manufacturer’s recommendations. Concentration values were reported as the means of at least three determinations.

### 2.8. Cytotoxicity Assays and LDH Detection

To compare the cytotoxic effects of the tribodies with those of the clinically validated mAbs on tumor cells co-cultured with human lymphocytes, MDA-MB231 and BT-549 tumor cells were plated in 96-well flat-bottom plates at a density of 1 × 10^4^ cells/well overnight at 37 °C; then, the lymphocytes were added (3:1 effector/target ratio) and the co-cultures were treated for 48 h with 100 nM of TR0304 or T0506 tribodies, Relatlimab, Atezolizumab, or Pembrolizumab mAbs, used as single agents or in combination for comparison. As negative controls, the co-cultured cells were analyzed in the absence of treatments or in the presence of a human-unrelated IgG used at the same concentration. The cell supernatants were collected at the end of incubation, and the level of lactate dehydrogenase (LDH) released by the cells was evaluated as a marker of tumor cell lysis and expressed as a percentage with respect to the max lysis obtained after treatment with 10% Triton X-100, following the manufacturer’s recommendations for the LDH detection kit (Thermofisher Scientific, Rockford, IL, USA) [18,19].

### 2.9. Cell Growth Inhibition Assays

To test the viability of tumor cells after treatments with the TR0304 bispecific tribody, the anti-PD-L1 and anti-LAG-3 clinically validated mAbs, or their combinations, MDA-MB-231 and BT-549 cells were plated in 96-well flat-bottom plates at a density of 1 × 10^4^ cells/well overnight at 37 °C and then treated with the indicated antibodies, used at concentrations of 100 and 200 nM, for 72 h. Untreated cells or cells treated with an unrelated IgG (100 nM) were used as negative controls. Cell survival was expressed as the percentage of viable treated cells with respect to untreated cells after cell counting via the trypan blue exclusion test [17,26].

### 2.10. Statistical Analyses

Error bars were calculated on the basis of the results obtained via at least three independent experiments. Analyses of activated lymphocytes were performed by using samples of hPBMCs obtained using at least three different donors. Statistical analyses were assessed using Student’s *t*-test (two variables). Statistical significance was established as *** *p* ≤ 0.001; ** *p* < 0.01; and * *p* < 0.05.

## 3. Results

### 3.1. The Binding of Bispecific Tribodies to Their Targets, Expressed on the Cell Surface or as Recombinant Proteins, in Comparison with the Clinically Validated mAbs

The novel tribodies, called TR0304 and TR0506, were previously generated in our laboratory by fusing a Fab derived from an anti-PD-L1 mAb (called PD-L1_1) and two scFvs derived from an anti-LAG3 mAb, called LAG-3_1, or by combining a Fab derived from LAG-3_1 mAb and two scFvs derived from an anti-PD-1 mAb (PD-1_1), respectively (see Figure 1A). These two novel human constructs should be endowed with more potent immunomodulatory activity than conventional monospecific mAbs, since they incorporate two different immune checkpoint binding domains, combine the binding specificities and biological properties of two different immunomodulatory mAbs in one single molecule, and, indeed, have already shown more potent antitumor effects with respect to their parental immunomodulatory antibodies [20]. Here, we decided to compare them to the clinically validated and used anti-LAG-3 (Relatlimab), anti-PD-L1 (Atezolizumab), and anti-PD-1 mAbs (Pembrolizumab), alone or in combination, for the treatment of different types of tumors [13,14,15,17,18,19,27,28,29].

**Figure 2:**
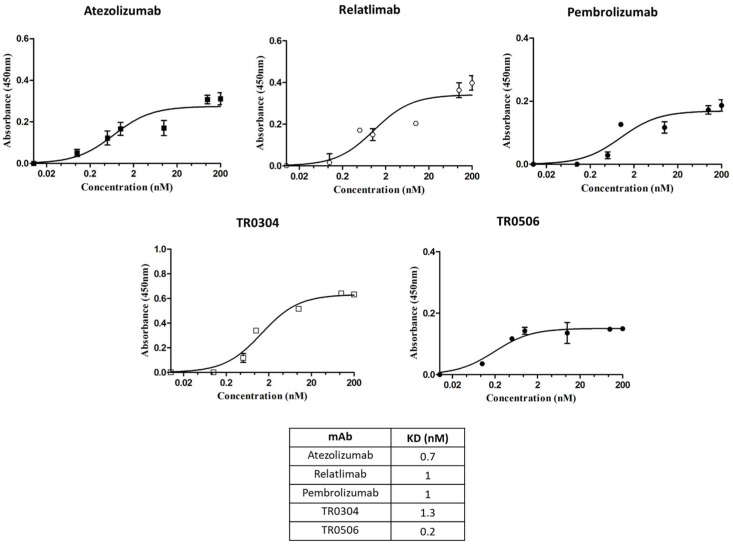
Binding to human lymphocytes of the tribodies TR0304 and 0506. Cell ELISAs to test the binding of the indicated TRs or validated antibodies to their targets expressed on activated lymphocytes. Binding curves were obtained by testing the bispecific constructs at increasing concentrations on human lymphocytes activated with anti-CD-3/CD-28 beads. The signals were detected by using the anti-Fab-HRP-conjugated secondary Ab. The values were reported as the means of at least three determinations from three different experiments. Error bars depict means ± SD.

The novel tribodies were first compared to the clinically validated mAbs by testing their ability to bind to their targets, expressed either in their native conformation on human-activated lymphocytes or as recombinant chimeric proteins. As reported in Figure 2, parallel cell ELISAs were performed by testing the commercial bivalent antibodies and the bispecific tribodies at increasing concentrations on lymphocytes after activation for 72 h with anti-CD-3/CD-28 beads [16]. In these assays, we tested novel bispecific compounds in comparison with conventional monospecific mAbs, even though the antibodies used are bivalent in a similar fashion to some of the tribodies, which are bivalent for one target and monovalent for the second one.

As reported in the table of Figure 2, the two novel TRs showed binding affinity to the cell surface targets in a sub or low-nanomolar range, comparable or even better than that of clinically validated mAbs.

**Figure 3:**
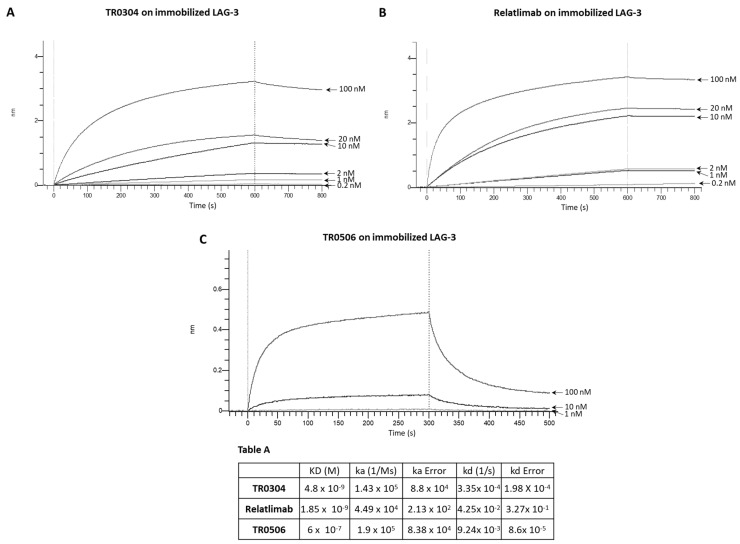
Binding kinetics of the bispecific TR0304 and TR0506 tribodies, compared to those of validated anti-LAG-3 mAb Relatlimab, on immobilized LAG-3 protein via BLI analyses. The sensorgrams reported in (**A**) for TR0304, (**B**) for Relatlimab, and (**C**) for TR0506 were obtained via BLI analyses. The recombinant LAG-3/Fc was used as ligand, whereas TR0304, TR0506, or Relatlimab was used as analyte and tested at increasing concentrations (0.2–100 nM). The sensorgrams show association and dissociation rates of the analytes. Table A reports the KD values and kinetics of the binding of the analytes to the immobilized ligand on a Protein A (proA) biosensor, processed according to the indicated formula, where A represents the analyte and B represents the immobilized ligand. ka and kd are the association and dissociation rate constants [30]. The data were analyzed using Octet Analysis Studio 13.0 Software (Sartorius, Fremont, CA, USA).

**Figure 4:**
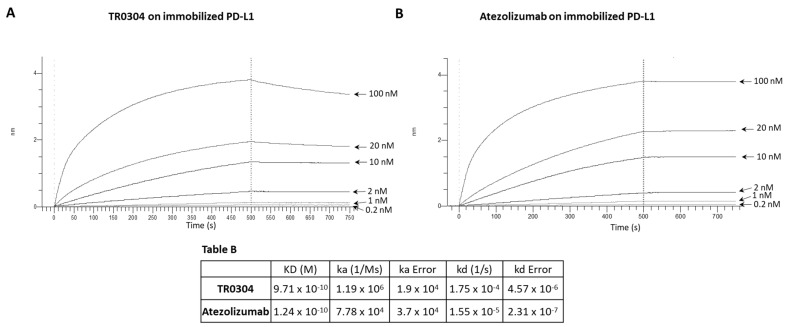
Binding kinetics of the bispecific TR0304 tribody, compared to those of anti-PD-L1 Atezolizumab, on immobilized PD-L1 protein via BLI analysis. The sensorgrams reported in (**A**) for TR0304 and (**B**) for Atezolizumab were obtained via BLI analyses. The recombinant PD-L1/Fc was used as ligand, and TR0304 or Atezolizumab was used as analyte and tested at increasing concentrations (0.2–100 nM). The sensorgrams show the rate of association and dissociation of the analytes. Table B reports the KD values and kinetics of the binding of the analytes to the immobilized ligand on a proA biosensor.

**Figure 5:**
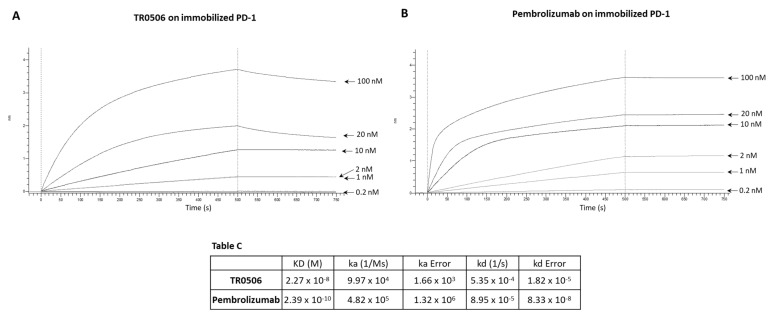
Binding kinetics of the bispecific TR0506 tribody, compared to that of anti-PD-1 Pembrolizumab, on immobilized PD-1 protein via BLI analyses. The sensorgrams reported in (**A**) for TR0506 and (**B**) for Pembrolizumab were obtained via BLI analyses carried out using the recombinant PD-1/Fc as the ligand, whereas TR0304 or Atezolizumab was used as analyte and tested at increasing concentrations (0.2–100 nM). The sensorgrams show the rates of association and dissociation of the analytes. Table C reports the KD values and kinetics of the binding of the analytes to the immobilized ligand on a proA biosensor.

We decided to further compare the binding properties and kinetics of TR0304 and TR0506 with the clinically validated mAbs by using a real-time methodology based on Biolayer Interferometry (BLI). To this aim, we immobilized the human recombinant chimeric LAG-3-Fc (Figure 3), PD-L1-Fc (Figure 4), or PD-1-Fc (Figure 5) proteins, used as ligands, on the surface of biosensor protein A (proA); then, we added each antibody specific for its target at increasing concentrations as the analyte. We used these assay conditions (by immobilizing on the biosensor the recombinant target protein and not the antibody, as it would be necessary to measure the monovalent 1:1 binding affinity) as this system better mimics that of the in vivo environment. Indeed, the circulating bivalent antibodies in the blood (comparable to flowing analytes in the assay) should bind to the targets exposed on the surface of immune cells (comparable to the immobilized ligand of the BLI assay).

Since TR0304 was composed of a Fab derived from an anti-PD-L1 mAb and two scFvs derived from an anti-LAG3 mAb [20], it was tested on immobilized LAG-3 (Figure 3) or PD-L1 (Figure 4) protein in comparison with the validated anti-LAG-3 Relatlimab or anti-PD-L1 Atezolizumab, respectively, whereas TR0506, composed of a Fab derived from the same anti-LAG3 mAb and two scFvs derived from an anti-PD-1 mAb [20], was tested on immobilized LAG-3 (Figure 3) or PD-1 (Figure 5) protein in comparison with Relatlimab or the validated anti-PD-1 Pembrolizumab, respectively.

As shown in Figure 3, Figure 4 and Figure 5, they bound to their targets in a dose-dependent fashion. Tables A–C indicate the values obtained via the sensorgrams, showing the KD values and the kinetics of binding association (ka) and dissociation (kd) of the single analytes to their respective immobilized LAG-3 (Table A), PD-L1 (Table B), or PD-1 (Table C) ligand on the proA biosensors [30].

The results show that TR0304 is able to bind to both its targets with an affinity comparable to that of the clinically validated mAbs, as indicated by the similar KD values. On the other hand, TR0506 showed high affinity for PD-1 and lower affinity to LAG-3 when compared to the clinically validated Relatlimab. The latter result can be explained by considering that TR0304 contains two scFvs recognizing LAG-3 (bivalent), whereas TR0506 is monovalent for LAG-3, having only a single Fab arm specific for LAG-3 with respect to the bivalent Relatlimab.

### 3.2. Epitope Analysis via BLI

**Figure 6:**
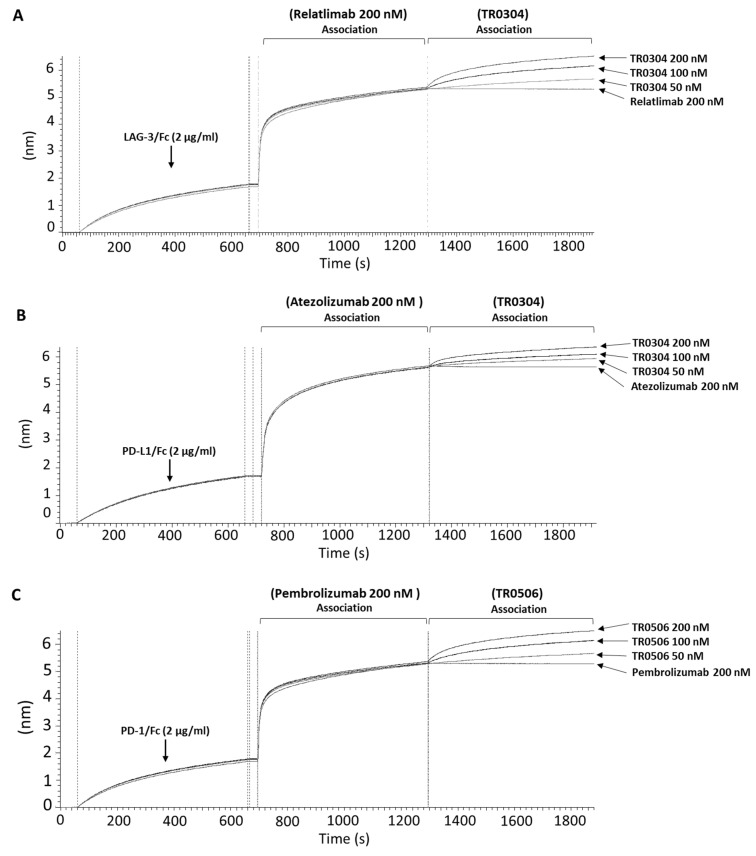
Competitive binding of TR0304 and TR0506 tribodies with that of the clinically validated mAbs specific for the same targets. The compounds were tested in parallel on immobilized PD-L1, LAG-3, or PD-1 recombinant proteins via BLI analyses. Sensorgram (**A**) shows the binding of the TR0304 construct following saturation of the sensor with Relatlimab at a concentration of 200 nM. Sensorgram (**B**) shows testing of TR0304 at three increasing concentrations (50, 100, and 200 nM) after the saturation with Atezolizumab (200 nM). Sensorgram (**C**) shows the binding of the TR0506 tribody at the same concentrations, after saturating the sensor with Pembrolizumab at a concentration of 200 nM. For each sensorgram, a second incubation is also shown with 200 nM of the same antibody, used as primary analyte, to demonstrate the saturation of the antigen epitope (negative control).

In order to investigate the epitopes recognized by these novel constructs and, in particular, to verify whether these motifs were different from those recognized by the monoclonal antibodies already approved for clinical use, epitope binning analyses were performed via BLI. For this purpose, the following recombinant proteins were immobilized as ligands on the proA biosensors: LAG-3/Fc protein (Figure 6A), PD-L1/Fc protein (Figure 6B), and PD-1/Fc protein (Figure 6C). Each ligand was then saturated with two consecutive incubations of each specific validated monoclonal antibody (Relatlimab for LAG-3, Atezolizumab for PD-L1, and Pembrolizumab for PD-1) used at a concentration of 200 nM; then, when a plateau was reached, the tribodies (TR0304 for LAG-3 and PD-L1; TR0506 for PD-1) were added at the indicated increasing concentrations. As negative controls, the same antibodies previously used to saturate the ligand immobilized on the sensor were added for a second time in parallel assays at the highest concentration (200 nM) to guarantee that the biosensor was fully saturated and no additional binding could occur in the event of an antibody recognizing the same antigen epitope. As shown in the sensorgrams of Figure 6, the tribodies were still able to bind to their targets, even when they were previously saturated with the respective clinically validated mAbs, thus suggesting that the tribodies recognized different epitopes from those of the validated antibodies, even though we cannot exclude the possibility that they could be partially overlapping, considering the lower signal observed with respect to that obtained in the absence of saturating mAb.

### 3.3. Effects on the Activation of hPBMCs (Cytokine Secretion) of Bispecifics with Respect to Combinations of Validated mAbs

Once we confirmed the binding specificity and affinity, we tested the ability of the two novel tribodies, TR0304 and TR0506, to activate human lymphocytes by measuring the levels of IL-2 and IFNγ cytokines secreted in the supernatant. To this aim, we stimulated human peripheral blood lymphocytes (hPBMCs) with Staphylococcal Enterotoxin B (SEB) and treated them for 66 h with TR0304 or TR0506, in comparison with the clinically validated mAbs Atezolizumab, Pembrolizumab, or Relatlimab used as single agents or in appropriate combinations at a concentration of 60 nM. As negative controls, untreated lymphocytes and hPBMCs stimulated with SEB alone or treated with an unrelated human IgG mAb were used in parallel assays.

**Figure 7:**
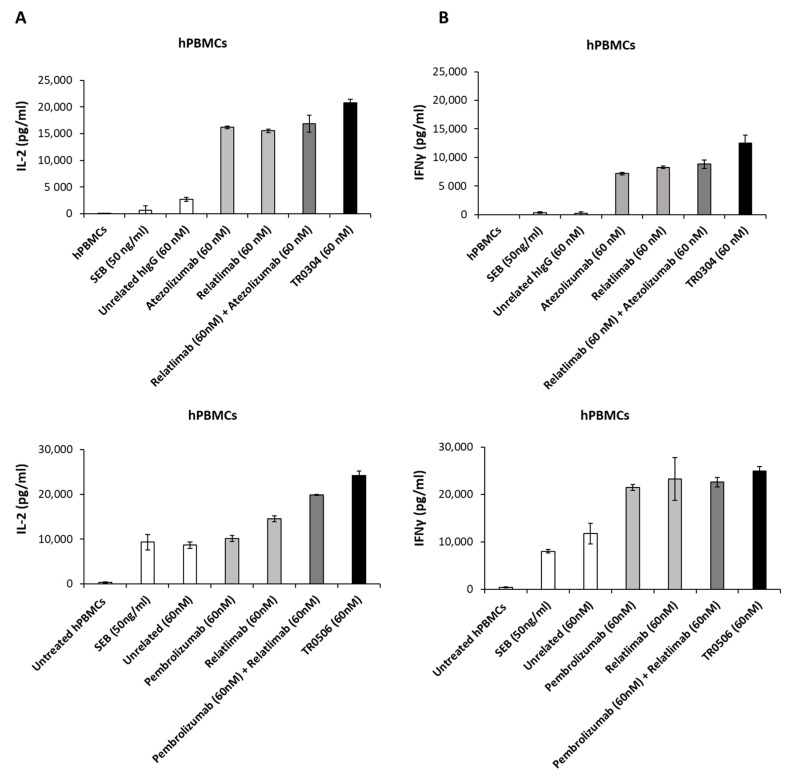
Effects of anti-PD-L1, anti-PD-1, or anti-LAG-3 validated mAbs compared with those of TR0304 or TR0506 on hPBMC activation. hPBMCs were incubated with SEB for 66 h, in the absence or in the presence of the clinically validated Atezolizumab, Pembrolizumab, or Relatlimab used as single agents (light gray bars) or in combination (dark gray bars), or the novel TR0304 or TR0506 tribody (black bars), at the indicated concentrations. As negative controls, untreated hPBMCs and hPBMCs treated with SEB alone or with an unrelated mAb (IgG4 or IgG1, which showed the same result) were used (white bars). IL-2 (**A**) and IFNγ (**B**) levels were evaluated via ELISAs.

As shown in Figure 7, the novel tribodies were more efficient in inducing hPBMC activation than the mAbs in clinical use either used alone or in combination, showing higher secretion levels of both IL-2 and IFNγ. In particular, the concentrations of IL-2 reached 20,775 pg/mL and 24,218 pg/mL in TR0304 and TR0506 treatments, respectively, which are almost 20% higher than the treatments based on the combination of the clinically validated mAbs (Figure 7A), even though the concentration of the TR (60 nM) corresponds to half of the total concentration of the combinatorial treatments (120 nM). Similarly, IFNγ secretion reached concentrations of 12,500 pg/mL and 25,000 pg/mL in the supernatant when the hPBMCs were treated with TR0304 and TR0506, respectively, representing increases of 10% and 30% with respect to the levels obtained with the corresponding combinations of the mAbs in clinical use (Figure 7B).

### 3.4. The Effects on Tumor Cell Cytotoxicity in Co-Culture-Based Assays with hPBMCs of Bispecifics with Respect to Combinations of Validated mAbs

Considering the ability of TR0304 and TR0506 to induce the activation of lymphocytes more efficiently than Relatlimab, Atezolizumab, or Pembrolizumab, either when used alone or in combination, we decided to also compare their cytotoxic effects on tumor cells used alone or in co-cultures with immune cells with those of these mAbs that are currently in clinical use for the treatment of melanoma, lung, and breast cancer [13,14,15].

We first tested the effects of the anti-PD-L1-specific TR0304 on tumor cells expressing high levels of PD-L1 in comparison with the combination of Atezolizumab and Relatlimab. As shown in Supplementary Figure S1, TR0304 inhibited the tumor growth of triple-negative breast cancer cells (MDA-MB-231 and BT549 cells) more efficiently than the combination of the validated mAbs, particularly when used at a concentration of 200 nM.

**Figure 8:**
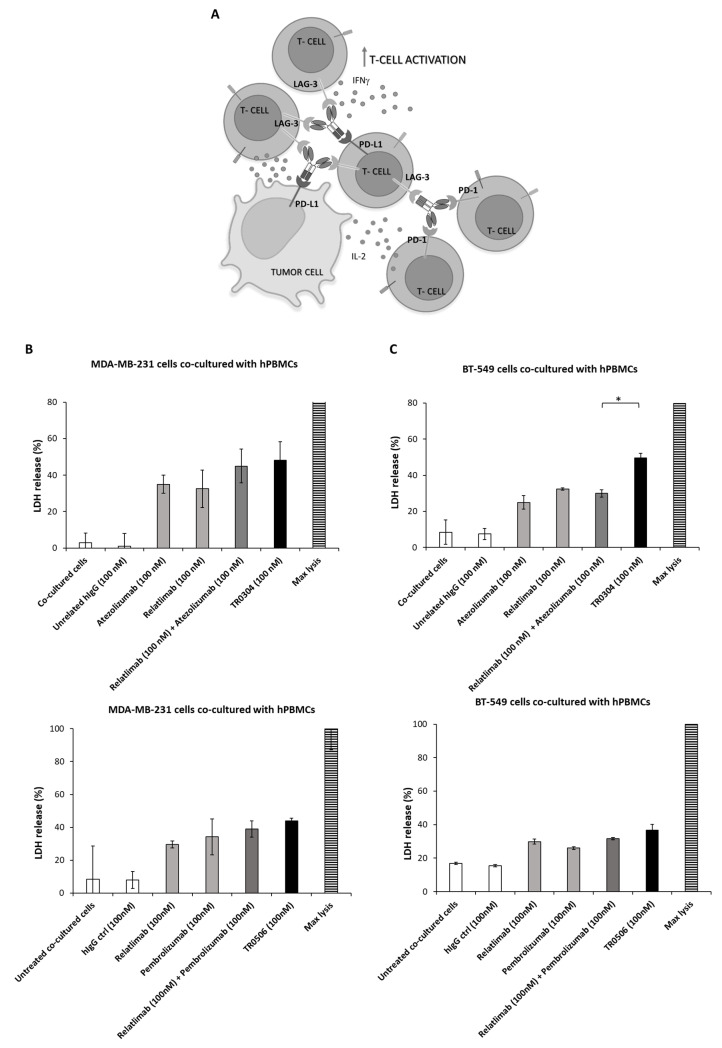

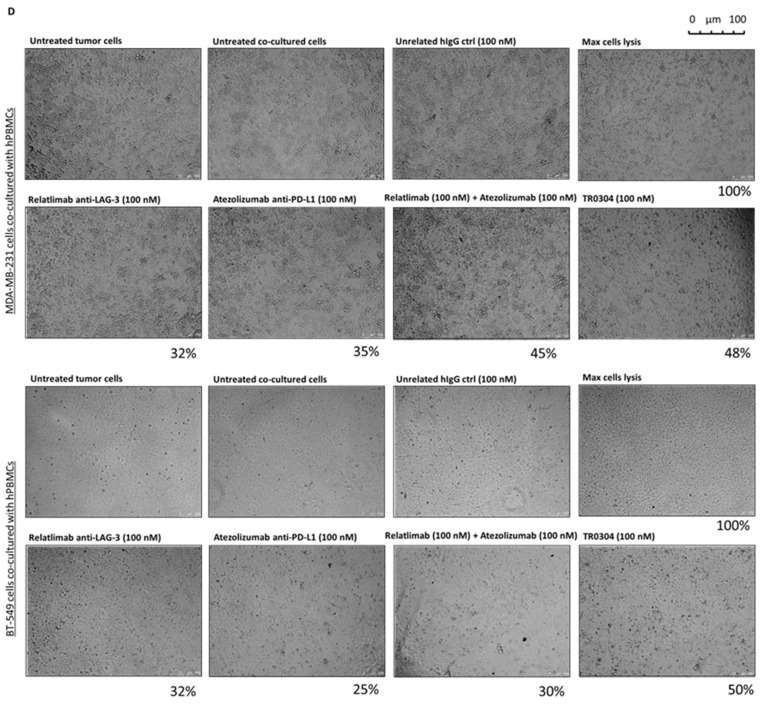
Cytotoxic effects of TR0304 or TR0506 on two different breast cancer cell lines co-cultured with hPBMCs. Schematic representation of the co-culture-based assays used for testing the effects of TRs (**A**). MDA-MB-231 (**B**) or BT-549 (**C**) tumor cells were cultured with hPBMCs (3:1 effector/target cell ratio) and treated for 48 h with the clinically validated anti-PD-L1, anti PD-1, or anti-LAG-3 mAbs used as single agents (light gray bars) or in combination (dark gray bars), with respect to the novel TR0304 or TR0506 tribodies (black bars), at the indicated concentrations. Untreated cells or cells treated with an unrelated mAb (white bars) were used as negative controls. The supernatants of the co-cultures were collected and the LDH levels were measured in order to evaluate the cell lysis induced by the treatments. The values were expressed as percentage and compared to the max cell lysis (striped bars) obtained after incubating the cells with 10% Triton X-100 for 20 min. Cells untreated or treated with an unrelated IgG4 or IgG1 were used as negative controls and showed the same result. Error bars depict means ± SD. *p*-values for the indicated compounds are * *p* < 0.05. (**D**) The images were obtained via Leica Advanced microscopy after hPBMC removal. Scale Bar is reported in the figure.

We further investigated the cytotoxic effects of the TRs when MDA-MB-231 (Figure 8B,D), BT-549 (Figure 8C,D), or A-549 lung cancer cells [17,19] were co-cultured with human PBMCs (3:1 effector/target ratio) in 96-well plates and treated with the tribodies, or the corresponding validated mAbs, used as single agents or in combinatorial treatments, at a concentration of 100 nM for 48 h at 37 °C. As negative controls, unrelated IgG1 and IgG4 were used in parallel assays. After incubation, cell lysis was evaluated by measuring lactate dehydrogenase (LDH) released in the cell supernatants. As shown in Figure 8, the novel bispecific tribodies (black bars) induced about 50% tumor cell lysis at a concentration of 100 nM, comparable to or even better than that obtained with the combination of the validated mAbs (dark gray bars) used at a doubled concentration (100 + 100 nM of each mAb).

Considering the highest antitumor efficacy of the tribodies, we also analyzed the levels of inflammatory cytokines released in the supernatants of these co-cultures to confirm these encouraging results.

**Figure 9:**
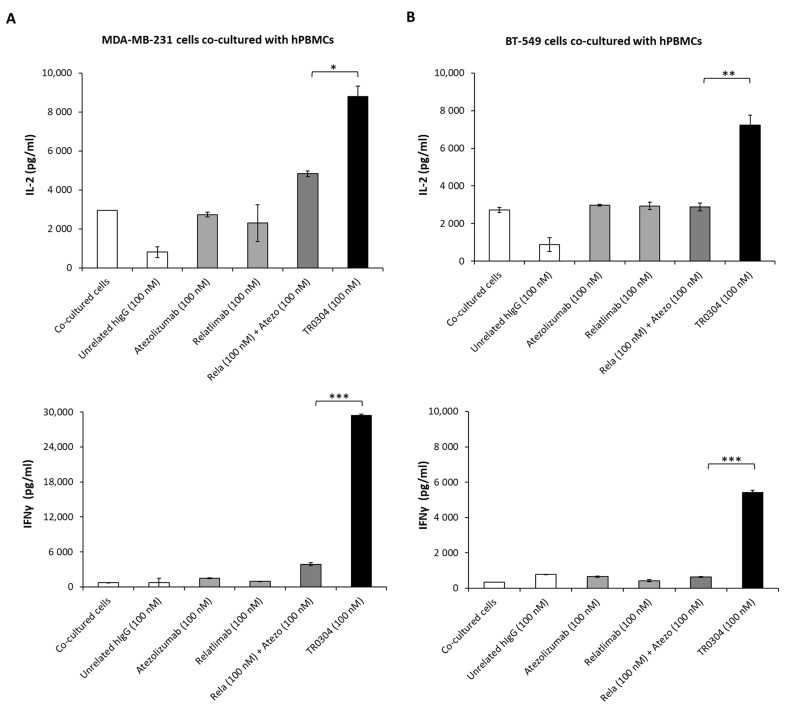
Secretion of cytokines induced by treatment with TR0304. The effects of the TR0304 tribody on hPBMC activation in co-cultures with MDA-MB231 (**A**) or BT-459 (**B**) tumor cells were also analyzed by measuring IL-2 and IFNγ secretion levels by using ELISA kits [16,17] on supernatants collected after the indicated treatments. Cells untreated or treated with an unrelated IgG1 or IgG4 were used as negative controls and showed the same result, represented by a single IgG bar. Error bars depict means ± SD. *p*-values for the indicated compounds are *** *p* ≤ 0.001; ** *p* < 0.01; and * *p* < 0.05.

As shown in Figure 9, the novel tribody TR0304 induced a higher secretion of IL-2 and IFNγ than the corresponding combination of the mAbs in clinical use. In particular, when the tumor cells were co-cultured with hPBMCs and treated with TR0304 at a concentration of 100 nM, the secretion of IL-2 in the MDA-MB-231 and BT-549 co-cultures was, respectively, 45% and 60% higher (8780 pg/mL and 7230 pg/mL) when compared with the combination of Relatlimab + Atezolizumab (100 nM + 100 nM) (Figure 9A,B upper panels). Surprisingly, the improvement in the secretion of IFNγ when the co-cultures were treated with TR0304 was even higher, being about 88% stronger than the treatment with the combination of clinically validated mAbs (Figure 9A,B lower panels).

## 4. Discussion

Cancer immunotherapy has engendered a major improvement in patients’ quality of life and survival. In particular, in recent years, significant progress has been achieved with the success of chimeric antigen receptor (CAR) T cell therapy and bispecific antibodies, such as Blinatumomab, for hematological malignancies, leading to the approval of a number of CD19-targeted therapy products [31,32] by the US Food and Drug Administration (FDA). The first clinical trials mainly included leukemia and lymphoma, but solid tumors are now becoming the main challenge. Despite these promising results, the success of cancer immunotherapy in solid tumors has been limited due to several barriers. Indeed, a solid tumor is a complex tissue consisting of stromal cells, inflammatory cells, regulatory T cells (Tregs), and extracellular matrices (ECMs) that create a tumor microenvironment (TME), which often prevents effective lymphocyte priming, reduces lymphocyte infiltration, and inhibits T cell cytotoxic activity, thus leading to the failure of the host to effectively attack tumors [4,5].

Among the mechanisms of resistance to immunotherapy, including low infiltration of immune cells into tumor sites, an absence of antigenic proteins or defects in antigen presentation, and an inhibitory microenvironment, the expression of multiple immune checkpoints has recently been considered a critical one [21,22]. Indeed, the compensatory proliferation of Tregs due to incomplete depletion by checkpoint inhibitors and the upregulation of alternative checkpoint molecules play a key role in resistance to immunotherapy. Therefore, in solid tumors, immunotherapy will likely require the use of combinations of Immune Checkpoint Inhibitors (ICIs) that help to overcome the immunosuppressive TME and convert a “cold” tumor into a “hot” one. Combinatorial therapies have been successfully developed, such as the combination of anti-PD-1 and anti-CTLA-4 antibodies [33], which have resulted in longer progression-free survival and a higher rate of response than those observed with single mAbs.

More recently, the clinical activity of combinations has also been established by using a LAG-3-blocking mAb (Relatlimab) plus PD-1 mAb (Nivolumab) combination [34,35,36,37], and a number of LAG-3-targeting approaches are being investigated in clinical trials [34].

Ideally, a single molecule should be designed that is able to combine antibodies targeting two different ICs in order to increase the antitumor potency. This goal can be achieved with the construction of bispecific antibodies (BsAbs). Most developed BsAbs bind with an arm to a tumor-specific antigen and, with the second one, bind to immune cell receptors such as CD3 or CD16 to stimulate the immune response against cancer cells [23,24]. Three BsAbs are currently clinically approved and marketed, and more than 85 clinical trials targeting different tumor-associated antigens (TAAs) are in progress, demonstrating the validity of this approach.

Here, we fully characterized two novel bispecific tribodies belonging to a new generation of bispecifics, which are different from previous bispecifics targeting TAAs as they simultaneously target two ICs with synergistic activity such as PD-1/PD-L1 and LAG-3. In particular, the bispecific tribody TR0304, made up of a Fab derived from PD-L1_1 anti-PD-L1 mAb and two scFvs derived from LAG-3_1 anti-LAG-3 mAb [16,26], was tested for its binding properties and ability to activate lymphocytes in comparison with Relatlimab plus Atezolizumab, and TR0506, made up of a Fab derived from the same anti-LAG-3 mAb and two scFvs derived from an anti-PD-1 mAb, was tested in comparison with the combination of Relatlimab and Pembrolizumab.

We found that the two novel TRs showed comparable binding affinity to their targets with respect to validated mAbs, even though they recognize distinct or only partially overlapping epitopes. When tested for their functional properties, they showed an increased ability to induce lymphocyte activation and stronger in vitro cytotoxicity against tumor cells compared to combinatorial treatments of clinically validated mAbs.

Considering that tribodies also have other advantages with respect to combinatorial treatments, such as reduced production costs and lower dose requirements, we think that these novel immunomodulatory TRs could be used for therapeutic applications, particularly in monotherapy-resistant cancer patients.

## 5. Conclusions

We tested the binding and biological effects of novel TR0304 and TR0506 tribodies in comparison with the mAbs that target the same ICs and are currently approved for clinical use. The results showed that these new constructs bind to their targets with comparable or even higher affinity (when tested on hPBMCs) than the clinically validated mAbs, and recognize distinct or only partially overlapping epitopes with respect to those of Relatlimab, Atezolizumab, and Pembrolizumab.

More interestingly, the tribodies were able to activate hPBMCs, and subsequently induce tumor cell lysis in co-cultures of malignant and immune cells, with comparable or even higher efficiency than treatment with the combination of clinical mAbs. Considering the other advantages of TRs with respect to combinatorial treatments, such as reduced production costs and lower dose requirements, these novel immunomodulatory TRs could be used for therapeutic applications, particularly in monotherapy-resistant cancer patients.

## Data Availability

The data presented in this study are available in this article and Supplementary Material.

## Supplementary Materials

The following supporting information can be downloaded at: https://www.mdpi.com/article/10.3390/cancers15225345/s1, Figure S1: Anti-tumor effects of TR0304 on breast cancer cells compared to the clinically validated mAbs or their combinations.
