# Supplementary material for: A Comparison of the Antitumor Efficacy of Novel Multi-Specific Tribodies with Combinations of Approved Immunomodulatory Antibodies
Source: Cancers (Basel). 2023 Nov 9;15(22):5345. doi: 10.3390/cancers15225345 (PMC10670465; doi:10.3390/cancers15225345)
Supplement: Supplementary file 1 [file cancers-15-05345-s001.zip › cancers-2670070-supplementary.pdf]

Comparison of the anti-tumor efficacy of novel multi-specific Tribodies with combinations of approved immunomodulatory antibodies

Lorenzo Manna [1,2], Rosa Rapuano Lembo [2,3], Asami Yoshiyoka [4], Koji Nakamura [4], Margherita Passariello [1,2,*] and Claudia De Lorenzo [1,2,*]

[1] Department of Molecular Medicine and Medical Biotechnology, University of Naples "Federico II",
Via Pansini 5, 80131 Naples, Italy
[2] Ceinge—Biotecnologie Avanzate s.c.a.r.l., Via Gaetano Salvatore 486, 80145 Naples, Italy
[3] European School of Molecular Medicine, University of Milan, 20122 Milan, Italy
[4] Chiome Bioscience Inc., 3-12-1 Hommachi Shibuya-Ku, Tokyo 151-0071, Japan
* Correspondence: margherita.passariello@unina.it (M.P.); cladelor@unina.it (C.D.L.);
Tel.: +39-081-3737868 (C.D.L.)

Supplementary Figure and Legend

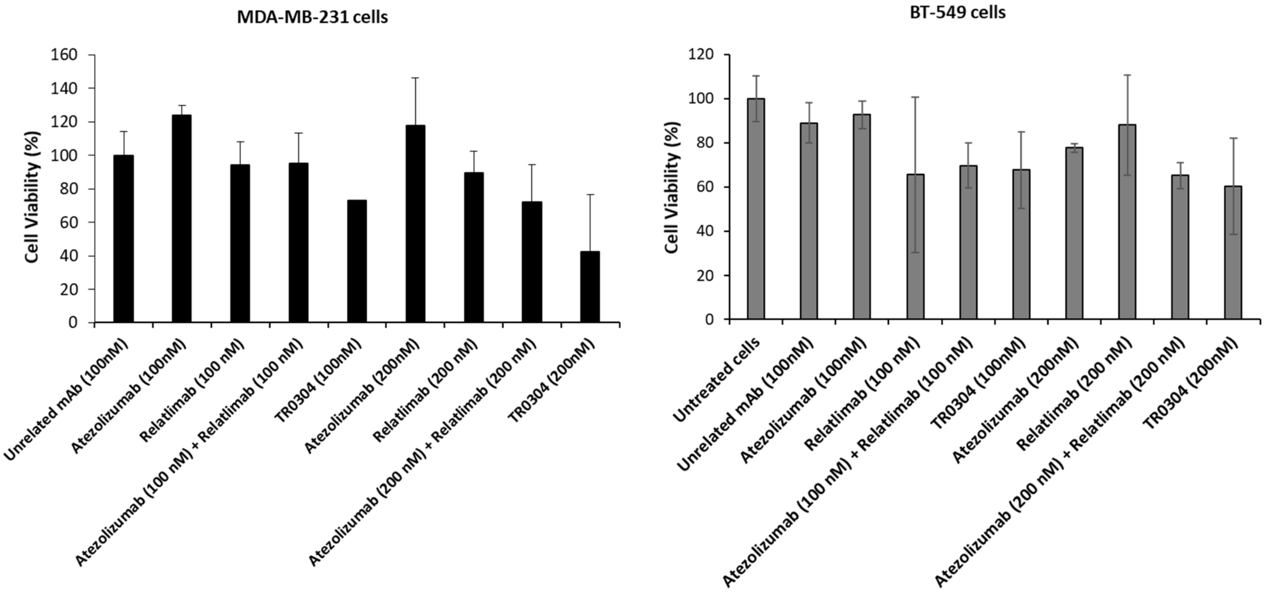

Supplementary Figure S1. Anti-tumor effects of TR0304 on breast cancer cells compared to the clinically validated mAbs or their combinations. MDA-MB-231 and BT-549 cells were incubated for 72 hours at 37°C with the TR0304 bi-specific tribody, the anti-PD-L1, anti-LAG-3 clinically validated mAbs or their combinations, at the indicated concentrations. Cell survival is expressed as percent of viable treated cells with respect to untreated cells. Error bars depicted means ± SD.
